# Evaluation of cytokines in the tumor microenvironment of lung cancer using bronchoalveolar lavage fluid analysis

**DOI:** 10.1007/s00262-020-02798-z

**Published:** 2021-01-04

**Authors:** Pascal Bezel, Alan Valaperti, Urs Steiner, Dieter Scholtze, Stephan Wieser, Maya Vonow-Eisenring, Andrea Widmer, Benedikt Kowalski, Malcolm Kohler, Daniel P. Franzen

**Affiliations:** 1 Department of Pulmonology, University Hospital Zurich, Raemistrasse 100, 8091 Zurich, Switzerland; 2 Department of Immunology, University Hospital Zurich, Gloriastrasse 23, 8091 Zurich, Switzerland; 3 Department of Pulmonology, City Hospital Triemli, Birmensdorferstrasse 497, 8063 Zurich, Switzerland; 4 Department of Pulmonology, City Hospital Waid, Tièchestrasse 99, 8037 Zurich, Switzerland

**Keywords:** Lung cancer, Tumor microenvironment, Cytokines, Bronchoalveolar lavage fluid, Whole-blood serum

## Abstract

**Introduction:**

Lung cancer is the leading cause of death by cancer. In recent years, immunotherapy with checkpoint inhibitors (ICI) emerged as a promising new therapeutic approach. However, a deeper understanding of the immunologic responses adjacent to the tumor known as tumor microenvironment (TME) is needed. Our study investigated TME of lung cancer by analyzing cytokines in bronchoalveolar lavage fluid (BALF).

**Materials and methods:**

Between January 2018 and June 2019, 119 patients were prospectively enrolled in this study. For each cancer patient, levels of 16 cytokines (fractalkine, granulocyte–macrophage colony-stimulating factor (GM-CSF), interferon gamma (IFN-γ), tumor necrosis factor alpha (TNF-α), and interleukins (IL): IL-1b, IL-2, IL-4, IL-5, IL-6, IL-7, IL-8, IL-10, IL-12p70, IL-13, IL-17A, and IL-23) were measured in BALF and serum and compared to healthy individuals and patients with other lung diseases.

**Results:**

There were several significant differences of cytokine levels of patients with lung cancer compared to healthy individuals. However, none of them remained in the multivariate analysis compared to other lung diseases in either BALF or serum. Furthermore, there were no significant differences between the groups in cell differentiation of either BALF or serum. Cytokine levels in BALF were generally near the lower detection limit and showed almost no correlation with their respective levels measured in serum of the same individual.

**Conclusions:**

Cytokines in BALF and serum of lung cancer patients may indicate unspecific inflammation. BAL is not recommendable as a tool to investigate TME of lung cancer. Therefore, cytokines measured in BALF are probably not appropriate as predictors in patients treated with ICIs.

**Supplementary Information:**

The online version contains supplementary material available at 10.1007/s00262-020-02798-z.

## Introduction

Lung cancer is the most common type of cancer worldwide and with 1.8 million reported cases of death in 2018 the leading cause of death by cancer in both men and women [1]. Its high lethality is mainly attributed to late appearance of symptoms leading to detection in already advanced tumor stages when curative treatment is usually no longer possible. In recent years, immunotherapy with checkpoint inhibitors (ICI) emerged as a promising new therapeutic approach in advanced or metastasized tumor stages, which may be able to revolutionize modern anticancer treatment [2, 3]. However, to develop new ICI or to choose the most promising ICI for a specific cancer phenotype, a deeper understanding of the immunologic responses adjacent to the tumor is needed. This so-called tumor microenvironment (TME) is formed by cancer cells, immune cells, stromal cells, and cytokines. Cytokines are immunomodulatory proteins, which are expressed by a variety of cells ranging from immune cells like macrophages and lymphocytes to endothelial cells and fibroblasts. During inflammation, they act as communicators between immune cells for regulating cell growth, maturation, and responsiveness [4, 5]. As recent data suggest a higher response rate to ICIs in “hot” or "active" tumors defined by immunological activation of the TME, there have been several trials trying to find biomarkers for either diagnosis or patient selection for ICI. However, so far, only the expression of programmed cell death ligand-1 (PD-L1) on cancer cells as well as a high tumor mutational burden has been found to show a positive predictive value for the efficacy of either PD-1 or PD-L1 inhibitors [6–12].

Bronchoalveolar lavage (BAL) is a sample technique of flexible bronchoscopy, which obtains liquid biopsy by flooding and subsequent aspiration of normal saline from the investigated lung segment or subsegment. The recovered fluid sample physiologically contains inflammatory cells and potentially tumor cells for cytological diagnostics. In addition, several biochemical elements acting as possible biomarkers may possibly be detected in BAL fluid (BALF). Although BAL drains a relatively large area surrounding a lung tumor and, therefore, the diagnostic yield is hypothetically not dependent on a bronchus leading to it, the diagnostic usefulness of BAL is unacceptably low with a reported sensitivity for the diagnosis of lung cancer of 29% when only cytological analyses are considered [13]. However, with the upcoming interest in ICIs, BAL gained new attention as a tool to access TME surrounding a lung cancer [14–23]. With our study, we aimed to investigate TME of lung cancer by analyzing various cytokines in BALF and thus answering three questions:

1.  Are there certain cytokines, which are upregulated in lung cancer patients compared to healthy individuals and to patients with other lung diseases?
2.  Does the local immunological response (measured in BALF) have a correlation with the systemic immunological response (measured in blood serum)?
3.  Is BAL a reliable tool to investigate TME as assessed by cytokines?

## Materials and methods

### Patients

This study is part of a prospective multicenter study aimed to establish a biobank (“*BALOTHEK*”) containing BALF and blood serum for the investigation of various lung diseases in patients in whom BAL was indicated as part of their routine clinical evaluation. Enrolled patients were retrospectively grouped according to their final diagnosis as confirmed by histology and finally allocated into four groups describing different lung diseases: lung cancer, sarcoidosis, interstitial lung disease (ILD), and drug-related pneumonitis. A fifth group consisted of patients who underwent bronchoscopy for assessment of chronic cough with a normal chest computed tomography (CT) finding (i.e., absence of consolidation, ground-glass opacity, nodule, mass, or interstitial changes) and without evidence of lung disease during a follow-up time of six months. Thus, the latter group served as healthy control group with structural normal lung parenchyma. Exclusion criteria for enrollment in *BALOTHEK* were history of lung transplantation, patient vulnerability such as pregnancy or emergency setting, as well as possible sampling and processing failures, such as a low BALF recovery rate resulting in less than 10 ml of BALF for the purpose of the biobank or a BAL-to-processing time exceeding 1 h as described elsewhere [24].

**Fig. 1:**
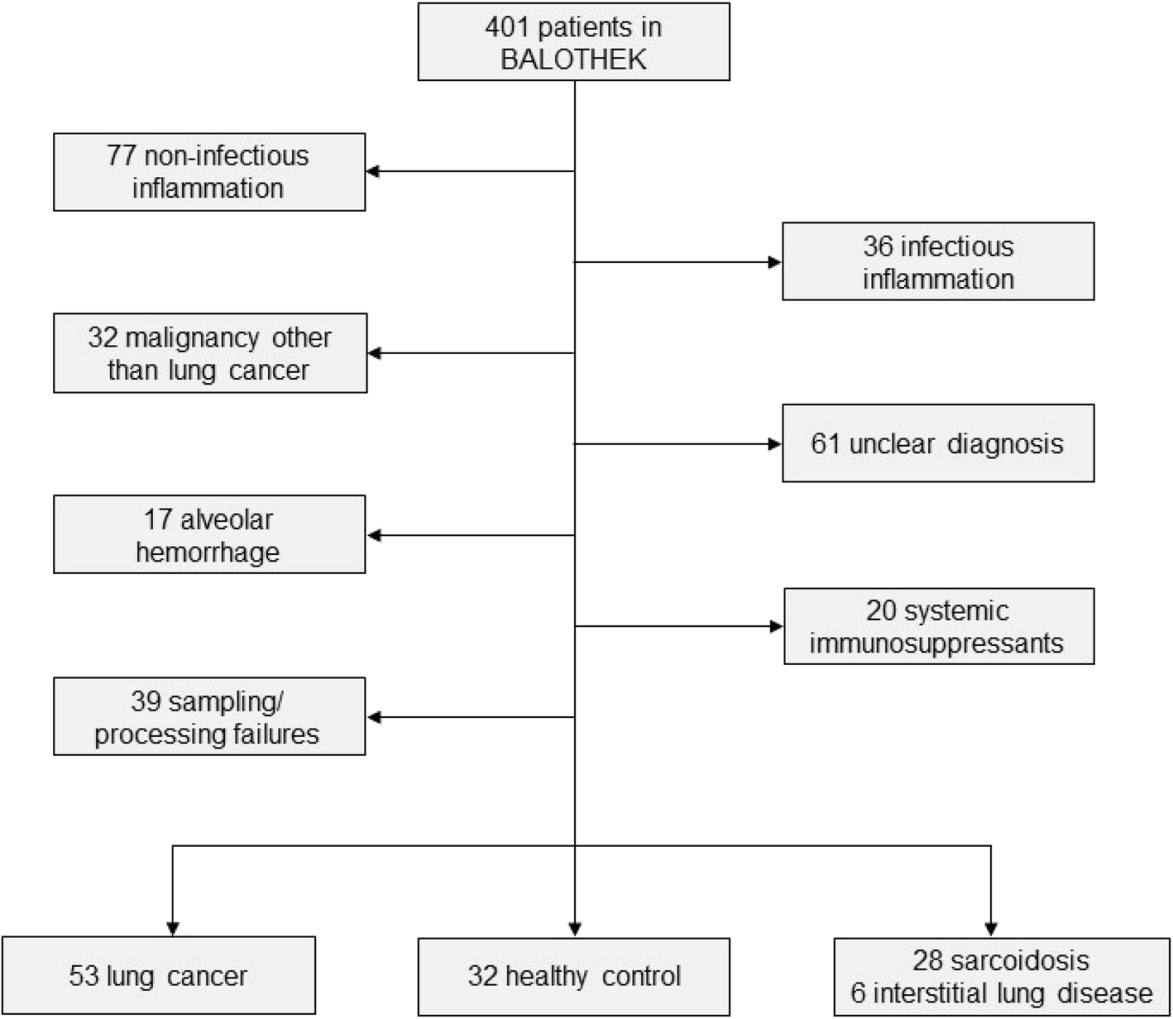
Selection of study population

Between January 2018 and June 2019, a total of 401 adult patients were prospectively enrolled in the *BALOTHEK*. After exclusion of patients with conditions possibly influencing the cytokine levels (infection, systemic immunosuppressants including systemic corticosteroids, and alveolar hemorrhage), uncertain diagnosis or malignancy other than lung cancer, and sampling/processing failures, 119 patients were included for the purpose of this study (Fig. 1).

All patient-related data including demographic and clinical data as well as bronchoscopy, radiology, and pathology reports were drawn from patient record files.

### Bronchoscopy and BAL technique

All patients underwent FB using Olympus (Olympus, Tokyo, Japan) bronchoscopes (190 series) under moderate sedation using propofol or general anesthesia according to the clinician’s decision. BAL was performed following the standardized procedure described by Baughman by injecting four portions of 50 ml (200 ml in total) of isotonic normal saline into the wedged segmental bronchus leading to the target lesion with the most prominent radiological finding [25]. BALF was recovered by gentle suction with the same syringe and collected in a graduated plastic cylinder. Approximately 50 ml of BALF was reserved for routine clinical analyses; excess fluid was used for the purpose of *BALOTHEK*.

### Processing of BAL fluid and blood specimens

BALF was collected in plastic tubes and centrifuged at 1′000 rounds per minute for 10 min at room temperature. The supernatant was collected and stored at − 80 °C for later analyses. The aliquots were only thawed once for analysis to prevent falsification of cytokine levels by repeated freezing and thawing. The routinely performed analysis of BALF for cell differentiation was performed by ADVIA 2120i (Siemens Healthcare AG, Zurich, Switzerland) via peroxidase staining. Cell differentiation included cell count, macrophages, lymphocytes, neutrophils, eosinophils, mast cells, and plasma cells. Blood samples were drawn as part of the routinely performed pre-interventional peripheral vein access. Whole blood was collected in a 10-ml BD Vacutainer Clot Activator Tube (CAT, Plus Blood Collection Tubes, Becton Dickinson, Plymouth, UK) and centrifuged at 3′500 rounds per minute for 10 min at room temperature. The subsequent process was analogous to the handling of BALF.

### Selection of cytokines

We specifically selected cytokines which were verified in previous studies as factors in TME of lung cancer, serving as rationale for their use in this study. Fractalkine is a prohibiting factor for metastasis and has a particularly high expression in the lungs [26]. Granulocyte–macrophage colony-stimulating factor (GM-CSF) can act pro-inflammatory as well as anti-inflammatory depending on the concentration and its environment [27, 28]. When secreted by tumor cells themselves, GM-CSF may lead to immune evasion for the tumor by promoting the development of myeloid suppressor cells [29]. Interferon gamma (IFN-γ) has been shown to be downregulated in progressive tumor disease as a sign for lower expression by natural killer cells [30]. Tumor necrosis factor alpha (TNF-α) is a marker for alveolar macrophage activity and plays a role in inhibiting carcinogenesis as well as angiogenesis [31]. Notably, it has been shown to be increased in exhaled breath condensate in patients with NSCLC in a previous study [32]. Interleukin (IL) 1b and IL-6 also act as markers for alveolar macrophage activity, and have been shown to be increased in BALF of lung cancer patients, with increase of IL-1b acting as a positive prognostic factor and increase of IL-6 acting as a negative prognostic factor for survival in lung cancer [33–35]. IL-2 has a strong effect on activation of natural killer cells [36]. Furthermore, it has been shown to be increased in exhaled breath condensate in patients with NSCLC [32]. IL-4 and IL-13 have been shown to have higher histopathological expressions of NSCLC in a previous study [37]. IL-5 had been shown to have an augmenting role in metastasis in lung cancer in mice models [38]. IL-7 has been shown to have an anti-apoptotic and thus pro-carcinogenic effect on lung cancer cells [39]. IL-8 is a chemoattractant for various immune cells as well as an inducer of angiogenesis [4]. Elevated levels of IL-8 have been shown to predict decreased survival in lung cancer [35]. Furthermore, rapid decrease of IL-8 levels during anti-PD-1 treatment correlated with treatment success [40]. IL-10 has an immunosuppressive effect and has been shown to be expressed by alveolar macrophages in TME of primary lung cancer. Increased levels of IL-10 positively correlated with tumor progression (size, metastasis, and poor histologic differentiation) [41]. IL-12 and in its activated form IL-12p70 are activators of natural killer cells as well as alveolar macrophages [4]. In mice models, knock-out of IL-12 induced spontaneous development of lung carcinomas [42]. Additionally, IL-12 and IFN-γ act as necessary mediators of anti-PD-1 treatment [43]. IL-13 is a marker of natural killer cell activity and been shown to be associated with progression and metastasis of lung cancer [44, 45]. IL-17 is a promotor of angiogenesis and cell proliferation as well as an inhibitor of apoptosis. As such, IL-17 has been shown to have a correlation with tumor progression and metastasis of lung cancer [46]. IL-23 suppresses the activity of B cells, T cells, and natural killer cells and thus promotes tumor progression and metastasis in lung cancer [30, 47].

### Cytokine analysis

The cytokine analysis was conducted using a cytokine multiple array on a Luminex 200 platform (Luminex Corporation, Austin, TX, USA) with a high sensitivity Milliplex kit (HSTCMAG-28SK-10, Merck Millipore, Darmstadt, Germany). The array included the following cytokines: fractalkine, granulocyte–macrophage colony-stimulating factor (GM-CSF), interferon gamma (IFN-γ), tumor necrosis factor alpha (TNF-α), and several interleukins (IL): IL-1b, IL-2, IL-4, IL-5, IL-6, IL-7, IL-8, IL-10, IL-12p70, IL-13, IL-17A, and IL-23. The acceptance criteria included the % coefficient of variation of the intra-assay, inter-assay, and replicates of low concentrated BALF, as well as the lower limit of detection, the lower limit of quantification, and the linearity.

### Statistical analysis

Levels of each cytokine of the case group (lung cancer) were analyzed against two control groups consisting of healthy individuals and patients with other lung diseases (sarcoidosis and ILD).

All statistical analyses were performed using SPSS Statistics for Windows 22.0 (IBM, Armonk, NY, USA). Normal distribution was assessed by the Kolmogorov–Smirnoff test. As all data were not normally distributed, data are reported as median ± interquartile range (IQR) or as percentages, as appropriate. Test for likeness was conducted by Pearson Correlation. Differences of means between the cohorts were calculated by Mann–Whitney *U* Test. Variables with *p* value < 0.1 were entered into a multivariate regression model. Minimum power level was set at 0.8. [48] The confidence interval (CI) was defined as 95%. *p* values of all outcomes were two-sided; a value less than 0.05 was considered statistically significant.

As the complete array of cytokines was analyzed in every patient, measurements of cytokine concentration with a median of 0.00 do neither imply an analytical error in cytokine measurement nor a reduction of patient samples but rather a cytokine concentration below the measurable detection limit in vivo, which in itself was considered an important finding. Consequently, we included all results in the statistical analysis to show the complete set of data in our study.

## Results

### Baseline characteristics

In total, 119 patients were included in this study. We split the study population into three groups: a first group with 53 patients with lung cancer (44.5%), a second group with 32 healthy individuals (26.9%), and a third group with 34 patients with other lung diseases (28.5%) consisting of 28 patients with sarcoidosis (23.5%) and six patients with ILD (5.0%). Baseline characteristics for each group are shown in Table 1. There were significant differences in age, pack years, smoking status, and BALF recovery rate. However, there were no significant differences between the groups in cell differentiation of either BALF or serum.

**Table 1:** Baseline characteristics of all groups

	Lung cancer		Healthy control		Other lung diseases		*p* value
Number	53 (44.5)		32 (26.9)		34 (28.6)		
Demography							
Age, years	66.0 (59.5–73.0)		54.5 (42.3–68.0)		49.5 (37.8–56.5)		0.013*
Male gender	28 (52.8)		18 (56.3)		20 (58.8)		0.855
Smoking							
Current smokers	13 (24.5)		7 (21.9)		5 (15.2)		0.577
Quitters	35 (66.0)		9 (28.1)		10 (29.4)		< 0.001*
Pack years	40.0 (15.0–51.3)		1.0 (0.0–16.3)		0.0 (0.0–14.3)		0.007*
Bronchoscopy							
Propofol, ml	690 (440–880)		400 (265–575)		640 (460–840)		0.143
BALF recovery, %	30.0 (20.0–40.0)		55.0 (42.5–62.0)		50.0 (43.8–60.0)		0.015*
BALF findings							
Cell count	49.3 (22.2–106.7)		107.5 (63.3–203.8)		123.0 (53.4–199.0)		0.458
Macrophages, G/l	43.4 (19.1–100.2)		77.8 (46.8–133.7)		83.0 (36.5–126.0)		0.421
Macrophages, %	88.0 (78.0–95.0)		91.5 (82.8–95.0)		66.8 (56.1–83.6)		0.256
Lymphocytes, G/l	1.8 (0.7–6.7)		5.0 (3.6–17.1)		22.9 (8.7–63.3)		0.510
Lymphocytes, %	3.3 (1.5–11.8)		6.0 (3.5–12.0)		27.8 (13.1–40.1)		0.115
Neutrophils, G/l	2.5 (0.6–6.3)		1.6 (0.9–7.9)		1.8 (0.5–5.7)		0.694
Neutrophils, %	4.8 (2.0–6.6)		2.0 (1.0–4.9)		1.8 (1.0–4.0)		0.279
Eosinophils, G/l	0.0 (0.0–0.0)		0.0 (0.0–0.7)		0.0 (0.0–0.0)		0.630
Eosinophils, %	0.0 (0.0–0.3)		0.0 (0.0–1.1)		0.0 (0.0–0.0)		0.606
Serum findings							
CRP, mg/dl	2.4 (1.0–23.5)		2.0 (1.0–5.5)		3.5 (1.2–16.5)		0.322
Leucocytes, G/l	7.7 (6.7–9.4)		6.9 (4.4–7.6)		6.0 (5.0–8.3)		0.517
Monocytes, G/l	0.6 (0.5–0.9)		0.5 (0.4–0.6)		0.5 (0.4–0.7)		0.960
Monocytes, %	8.4 (7.3–10.0)		8.2 (6.6–10.7)		8.9 (6.1–11.7)		0.319
Lymphocytes, G/l	1.5 (1.2–2.2)		1.6 (1.0–2.3)		1.2 (0.9–1.5)		0.254
Lymphocytes, %	21.2 (15.1–27.1)		26.9 (20.0–31.4)		20.0 (13.8–28.0)		0.273
Neutrophils, G/l	5.2 (4.0–6.8)		3.9 (2.6–4.7)		4.0 (2.8–6.4)		0.379
Neutrophils, %	68.5 (59.9–73.7)		59.9 (54.9–67.5)		68.0 (55.2–73.4)		0.571
Eosinophils, G/l	0.1 (0.1–0.2)		0.1 (0.0–0.3)		0.1 (0.1–0.2)		0.889
Eosinophils, %	1.4 (0.8–2.4)		2.2 (1.1–3.2)		1.8 (1.0–3.1)		0.383

* = *p* < 0.05. Data are presented as n (%) or median (IQR)

*BALF* bronchoalveolar lavage fluid, *CRP* C-reactive protein

Tumor-specific characteristics of the lung cancer group are shown in Table 2.

**Table 2:** Characteristics of lung cancer group

Type		
Adenocarcinoma	34	(64.2)
Squamous cell carcinoma	11	(20.8)
NSCLC	5	(9.4)
SCLC	3	(5.7)
Classification		
T		
1	14	(28.0)
2	19	(38.0)
3	12	(24.0)
4	5	(10.0)
N		
0	25	(49.0)
1	5	(9.8)
2	10	(19.6)
3	11	(21.6)
M		
0	34	(66.7)
1	17	(33.3)
Radiological findings		
Maximum diameter, mm	30.0	(18.0–46.0)
Tumor > 2 cm	40	(75.5)
SUVmax	9.6	(5.4–14.8)

Data are presented as *n* (%) or median (IQR)

*NSCLC* non-small cell lung cancer, *SCLC* small cell lung cancer, *SUVmax* maximum standardized uptake value

### Correlation of cytokines in BALF and serum

Statistical analysis of cytokine levels found in BALF compared to cytokine levels found in serum of all patients showed significant correlations only for IL-1b, as shown in Table 3.

**Table 3:** Correlation of cytokine levels in BALF compared to serum in all patients (*n* = 119)

Cytokine	BALF	Serum	*p* value	Pearson correlation coefficient *r*
Fractalkine	30.90 (0.00–77.81)	189.40 (131.84–282.94)	0.487	− 0.065
GM-CSF	6.97 (3.45–13.95)	25.72 (10.54–66.02)	0.357	0.086
IFN-γ	0.09 (0.00–0.27)	11.43 (4.91–23.14)	0.686	0.038
IL-1b	0.31 (0.23–0.57)	1.84 (0.36–3.14)	0.001*	0.303
IL-2	1.05 (0.53–1.92)	3.21 (1.53–5.61)	0.990	0.001
IL-4	0.00 (0.00–0.00)	22.16 (9.48–45.90)	0.425	− 0.075
IL-5	0.19 (0.12–0.31)	3.82 (2.25–5.69)	0.059	0.176
IL-6	1.12 (0.59–3.29)	4.74 (2.52–8.74)	0.635	0.044
IL-7	5.36 (2.15–11.11)	12.44 (9.62–18.43)	0.860	− 0.017
IL-8	33.34 (13.35–68.26)	10.60 (7.24–18.17)	0.500	0.063
Il-10	1.23 (0.36–3.27)	9.72 (5.38–14.90)	0.877	0.015
IL-12b	0.00 (0.00–0.05)	3.51 (1.60–6.65)	0.685	− 0.038
IL-13	0.00 (0.00–0.59)	5.39 (1.80–10.76)	0.570	− 0.053
IL-17a	0.22 (0.22–0.38)	11.82 (4.60–24.17)	0.708	0.035
IL-23	1.49 (0.00–3.48)	271.09 (142.92–492.15)	0.491	− 0.065
TNF-α	0.71 (0.31–1.47)	10.40 (5.71–14.46)	0.211	0.117

* = *p* < 0.05. Data are presented in median (IQR), cytokine levels in pg/ml

*BALF* bronchoalveolar lavage fluid, *GM-CSF* granulocyte–macrophage colony-stimulating factor, *IFN-γ* interferon gamma, *IL* interleukin, *TNF-α* tumor necrosis factor alpha

### Cytokines in BALF

The cytokine levels in BALF of lung cancer patients compared to both healthy individuals and patients with other lung diseases are shown in Table 4. According to univariate analysis, there were several significant differences in cytokine levels between both groups. As such, there was a striking increase of IL-8 in the lung cancer group (*p* = 0.002). However, after correction for co-factors (age, pack years, smoking status, and BALF recovery rate), none of the cytokines (including IL-8) showed statistically significant differences between the two groups. When subgroups were compared, multivariate analysis revealed significantly higher IL-8 levels in lung cancer patients compared to healthy subjects (*p* = 0.029) but not compared to patients with other lung diseases (*p* = 0.921) (data not shown). The subgroup analysis of different types of lung cancer and other characteristics listed in Table 2 showed no significant associations with cytokine levels in BALF.

**Table 4:** Cytokine levels in BALF of lung cancer patients compared to both healthy individuals and patients with other lung diseases

	Lung cancer	Non-lung cancer	Univariate analysis (*p* value)	Multivariate analysis (*p* value)
Fractalkine	19.53 (0.00–103.24)	37.02 (0.00–68.60)	0.615	0.518
GM-CSF	6.11 (0.30–18.82)	6.97 (4.08–11.72)	0.709	0.114
IFN-γ	0.09 (0.01–0.25)	0.01 (0.00–0.57)	0.543	0.762
IL-1b	0.37 (0.28–0.58)	0.28 (0.08–0.57)	0.006*	0.841
IL-2	1.47 (0.86–2.16)	0.75 (0.18–1.38)	< 0.001*	0.358
IL-4	0.00 (0.00–0.00)	0.00 (0.00–0.00)	0.019*	0.434
IL-5	0.19 (0.16–0.28)	0.19 (0.00–0.66)	0.396	0.389
IL-6	1.41 (0.64–5.30)	0.98 (0.53–2.52)	0.445	0.699
IL-7	4.18 (0.80–9.09)	6.94 (3.50–12.65)	0.006*	0.261
IL-8	48.85 (16.85–114.12)	17.71 (8.26–51.36)	0.002*	0.910
Il-10	0.78 (0.40–2.14)	1.98 (0.36–4.68)	0.317	0.596
IL-12b	0.04 (0.00–0.08)	0.00 (0.00–0.03)	< 0.001*	0.781
IL-13	0.00 (0.00–0.33)	0.24 (0.00–0.69)	0.048*	0.285
IL-17a	0.22 (0.22–0.22)	0.27 (0.22–0.73)	< 0.001*	0.309
IL-23	1.49 (0.00–1.49)	1.49 (0.00–21.44)	0.139	0.782
TNF-α	0.64 (0.14–1.28)	0.76 (0.42–1.63)	0.133	0.928

* = *p* < 0.05. Data are presented in median (IQR), cytokine levels in pg/ml

*BALF* bronchoalveolar lavage fluid, *GM-CSF* granulocyte–macrophage colony-stimulating factor, *IFN-γ* interferon gamma, *IL* interleukin, *TNF-α* tumor necrosis factor alpha

### Cytokine levels in serum

The cytokine levels in serum for patients with lung cancer compared to both healthy individuals and patients with other lung diseases are shown in Table 5. After correction for co-factors (age, pack years, smoking status, and BALF recovery rate), statistically significant increases of fractalkine, GM-CSF, IFN-γ, IL-1b, IL- 4, IL-8, IL-17a, IL-23, and TNF-α remained.

**Table 5:** Cytokine levels in serum in lung cancer patients compared to both healthy individuals and patients with other lung diseases

	Lung cancer	Non-lung cancer	Univariate analysis (*p* value)	Multivariate analysis (*p* value)
Fractalkine	239.28 (183.11–304.15)	149.85 (102.90–239.22)	< 0.001*	0.006*
GM-CSF	53.98 (35.04–70.93)	13.13 (8.17–22.30)	< 0.001*	0.009*
IFN-γ	17.30 (10.08–32.89)	8.53 (3.51–19.88)	< 0.001*	0.028*
IL-1b	2.86 (1.95–4.00)	0.43 (0.23–1.92)	< 0.001*	< 0.001*
IL-2	4.52 (2.35–6.61)	1.85 (1.37–4.58)	0.001*	0.170
IL-4	38.45 (23.79–52.58)	12.20 (6.07–22.89)	< 0.001*	0.002*
IL-5	3.86 (2.44–5.65)	3.57 (2.25–5.78)	0.743	0.970
IL-6	6.28 (3.80–9.13)	3.22 (1.68–6.46)	< 0.001*	0.685
IL-7	15.15 (10.20–19.34)	12.10 (8.84–17.50)	0.117	0.733
IL-8	14.08 (9.76–20.82)	8.18 (6.54–14.76)	< 0.001*	0.018*
Il-10	11.72 (8.33–17.09)	8.30 (2.46–13.81)	0.003*	0.766
IL-12b	4.99 (3.27–8.33)	2.41 (1.24–4.45)	< 0.001*	0.676
IL-13	8.90 (4.18–13.38)	2.98 (1.28–7.74)	< 0.001*	0.713
IL-17a	16.19 (10.29–32.45)	6.80 (1.92–16.58)	< 0.001*	0.003*
IL-23	412.70 (203.21–741.72)	215.83 (101.15–383.18)	0.001*	0.006*
TNF-α	12.40 (10.40–16.58)	7.10 (3.71–11.46)	< 0.001*	0.033*

* = *p* < 0.05. Data are presented in median (IQR). Cytokine levels are denoted in pg/ml

*BALF* bronchoalveolar lavage fluid, *GM-CSF* granulocyte–macrophage colony-stimulating factor, *IFN-γ* interferon gamma, *IL* interleukin, *TNF-α* tumor necrosis factor alpha

To differentiate between unspecific activation of the immune system and cancer-specific elevation of cytokine levels, multivariate analysis was repeated with separated control groups as seen in ESM Tables 6 and 7. Table 6 shows cytokine levels in serum in patients with lung cancer compared to healthy individuals. After correction for co-factors (age, pack years, smoking status, and BALF recovery rate), statistically significant elevations for fractalkine, GM-CSF, IFN-γ, IL-1b, IL-2, IL- 4, IL-8, IL-12b, IL-17a, IL-23, and TNF-α remained. In contrast, there was no statistically significant difference of any cytokine in lung cancer patients compared to patients with other lung diseases (ESM Table 7).

Notably, multivariate analysis of cytokine levels in patients with other lung diseases compared to cytokine levels in healthy individuals showed similar findings as the lung cancer group with statistically significant elevations for IL-1b (*p* = 0.016), IL-4 (*p* = 0.017), and TNF-α (*p* = 0.004) as well as a trend towards elevated levels for fractalkine (*p* = 0.067), GM-CSF (*p* = 0.073), IL-2 (*p* = 0.076), and IL-23 (*p* = 0.073) (not shown in table).

## Discussion

The primary goal of our study was to investigate whether certain cytokines are locally (BALF) or systemically (serum) upregulated in patients with lung cancer compared to healthy subjects or to patients with other lung diseases. Possibly owed to a lack of correlation between cytokines measured in BALF compared to those measured in the serum, we found that several cytokines in the serum of lung cancer patients were significantly increased compared to healthy controls, but not in BALF. Furthermore, our study was not able to detect a statistically significant increase of any cytokine in either BALF or serum in patients with lung cancer compared to patients with other lung diseases. Thus, the investigated cytokines were only able to indicate unspecific activation of the immune system rather than a differentiation between entities. Notably, there was an insignificant, but striking increase of IL-8 in BALF in lung cancer patients. This increase might be linked to the role of IL-8 in angiogenesis in addition to its role in the immunological response [49].

The secondary goal of our study was to investigate whether there is a correlation between the local immunological response in BALF and the systemic immunological response in blood serum. The majority of the investigated cytokines showed no significant correlation between cytokine levels measured in BALF compared to cytokine levels in serum of the same individual. This weak correlation of cytokines in BALF and serum is partly explained by diluted concentrations in BALF, which was previously described by our group [24]. However, our data suggest that local and systemic immunological responses do not necessarily correspond.

The tertiary goal of our study was to investigate cytokines in TME as assessed by BAL. Due to the inevitable dilution during BAL, the cytokine levels in BALF were generally near the lower detection limit, which may be associated with lower accuracy of the essay. Thus, the dilution may pose a limitation for BAL as a tool for analysis of TME. Certainly, a current topic of interest in oncology is the investigation on predictors of ICIs. However, most patients in our study received several, not study-controlled forms of cancer therapy, rendering the available data invalid for statistical analysis. Thus, further studies for the evaluation of cytokines as prognostic markers for ICIs are needed. However, according to the present study, we cannot recommend BALF as vehicle for corresponding cytokine analyses.

The second control group consisting of other lung diseases was inherently heterogenous and thus not eligible for a deduction of conclusions as a target of investigation itself. However, we included the data to put the findings of the analysis between the lung cancer group and the healthy control group into perspective, as almost all similar studies lacked such comparison. This allowed us to approximatively differentiate between unspecific cytokine increase due to generalized immunological response and tumor-specific and subsequently clinically relevant cytokine increase.

The limitation of this study was the number of cases used for statistical analysis, as it was not high enough to conduct a subgroup analysis for different types of cancers (e.g., small cell lung cancer). At the same time, we were able to ensure a high quality in patient selection by applying strict exclusion criteria.

Furthermore, this study was not designed to evaluate the prognostic value of cytokines. Consequently, survival was greatly influenced by the patient’s and treating physician’s decision for or against various treatment options. Therefore, survival as a parameter for prognostic value of cytokine levels was not subject of the study.

## Conclusion

Cytokines in BALF and serum of lung cancer patients may indicate unspecific inflammation. BAL is not recommendable as a tool to investigate TME of lung cancer. Therefore, cytokines measured in BALF are probably not appropriate as predictors in patients treated with ICIs.

## Supplementary Information

Below is the link to the electronic supplementary material.

Supplementary file1 (DOCX 255 KB)

## Data Availability

The datasets generated during and/or analyzed during the current study are available from the corresponding author on reasonable request.

## Abbreviations

BAL: Bronchoalveolar lavage
BALF: Bronchoalveolar lavage fluid
CI: Confidence interval
CRP: C-reactive protein
CT: Computed tomography
GM-CSF: Granulocyte–macrophage colony-stimulating factor
ICI: Immunotherapy with checkpoint inhibitors
IFN-γ: Interferon gamma
IQR: Interquartile range
IL: Interleukin
ILD: Interstitial lung disease
NSCLC: Non-small cell lung cancer
PD-L1: Programmed cell death ligand-1
SCLC: Small cell lung cancer
SUVmax: Maximum standardized uptake value
TME: Tumor microenvironment
TNF-α: Tumor necrosis factor alpha

## References

[1] International Agency for Research on Cancer. Cancer burden rises to 18.1 million new cases and 9.6 million cancer deaths in 2018; 2018. https://www.iarc.fr/wp-content/uploads/2018/09/pr263_E.pdf

[2] Reck M, Rabe KF (2017). Precision diagnosis and treatment for advanced non-small-cell lung cancer. N Engl J Med.

[3] Horn L, Spigel DR, Vokes EE (2017). Nivolumab versus docetaxel in previously treated patients with advanced non-small-cell lung cancer: two-year outcomes from two randomized, open-label, phase III trials (CheckMate 017 and CheckMate 057). J Clin Oncol.

[4] Dinarello CA (2007). Historical insights into cytokines. Eur J Immunol.

[5] Akdis M, Aab A, Altunbulakli C (2016). Interleukins (from IL-1 to IL-38), interferons, transforming growth factor β, and TNF-α: Receptors, functions, and roles in diseases. J Allergy Clin Immunol.

[6] Mellman I, Coukos G, Dranoff G (2011). Cancer immunotherapy comes of age. Nature.

[7] Peters S, Reck M, Smit EF, Mok T, Hellmann MD (2019). How to make the best use of immunotherapy as first-line treatment for advanced/metastatic non-small-cell lung cancer. Ann Oncol.

[8] Bianco A, Perrotta F, Barra G, Malapelle U, Rocco D, de Palma R (2019). Prognostic factors and biomarkers of responses to immune checkpoint inhibitors in lung cancer. Int J Mol Sci.

[9] Galon J, Pagès F, Marincola FM (2012). The immune score as a new possible approach for the classification of cancer. J Transl Med.

[10] Aerts JG, Hegmans JP (2013). Tumor-specific cytotoxic T cells are crucial for efficacy of immunomodulatory antibodies in patients with lung cancer. Cancer Res.

[11] Bedognetti D, Ceccarelli M, Galluzzi L (2019). Toward a comprehensive view of cancer immune responsiveness: a synopsis from the SITC workshop. J Immunother Cancer.

[12] Chen DS, Mellman I (2017). Elements of cancer immunity and the cancer-immune set point. Nature.

[13] Bezel P, Tischler V, Robinson C (2016). Diagnostic value of bronchoalveolar lavage for diagnosis of suspected peripheral lung cancer. Clin Lung Cancer.

[14] Kwiecien I, Skirecki T, Polubiec-Kownacka M, Raniszewska A, Domagala-Kulawik J (2019). Immunophenotype of T Cells expressing programmed death-1 and cytotoxic T Cell antigen-4 in early lung cancer: local vs. systemic immune response. Cancers.

[15] Naumnik W, Płońska I, Ossolińska M, Nikliński J, Naumnik B (2018). Prognostic value of osteoprotegerin and sRANKL in bronchoalveolar lavage fluid of patients with advanced non-small cell lung cancer. Adv Exp Med Biol.

[16] Naumnik W, Panek B, Ossolińska M, Naumnik B (2019). B cell-attracting chemokine-1 and progranulin in bronchoalveolar lavage fluid of patients with advanced non-small cell lung cancer: new prognostic factors. Adv Exp Med Biol.

[17] Zikos TA, Donnenberg AD, Landreneau RJ, Luketich JD, Donnenberg VS (2011). Lung T-cell subset composition at the time of surgical resection is a prognostic indicator in non-small cell lung cancer. Cancer Immunol Immunother.

[18] Staal-van den Brekel AJ, Dentener MA, Drent M, Ten VG, Buurman WA, Wouters E (1998). The enhanced inflammatory response in non-small cell lung carcinoma is not reflected in the alveolar compartment. Respir Med.

[19] Naumnik W, Naumnik B, Niklińska W, Ossolińska M, Chyczewska E (2016). Clinical implications of hepatocyte growth factor, interleukin-20, and interleukin-22 in serum and bronchoalveolar fluid of patients with non-small cell lung cancer. Adv Exp Med Biol.

[20] Fracchia A, Ubbiali A, El Bitar O (1999). A comparative study on ferritin concentration in serum and bilateral bronchoalveolar lavage fluid of patients with peripheral lung cancer versus control subjects. Oncology.

[21] Brcic L, Stanzer S, Krenbek D (2018). Immune cell landscape in therapy-naïve squamous cell and adenocarcinomas of the lung. Virchows Arch.

[22] Kopiński P, Wandtke T, Dyczek A (2018). Increased levels of interleukin 27 in patients with early clinical stages of non-small cell lung cancer. Polish Arch Internal Med.

[23] Jakubowska K, Naumnik W, Niklińska W, Chyczewska E (2015). Clinical significance of HMGB-1 and TGF-β level in serum and BALF of advanced non-small cell lung cancer. Adv Exp Med Biol.

[24] Valaperti A, Bezel P, Vonow-Eisenring M, Franzen D, Steiner UC (2019). Variability of cytokine concentration in whole blood serum and bronchoalveolar lavage over time. Cytokine.

[25] Baughman RP (2007). Technical aspects of bronchoalveolar lavage: recommendations for a standard procedure. Semin Respir Crit Care Med.

[26] Korbecki J, Simińska D, Kojder K (2020). Fractalkine/CX3CL1 in neoplastic processes. Int J Mol Sci.

[27] Tomczak M, Tomczak E, Kleka P, Lew R (2014) Using power analysis to estimate appropriate sample size (21.): 195–206

[28] Uemura Y, Kobayashi M, Nakata H (2006). Effects of GM-CSF and M-CSF on tumor progression of lung cancer: roles of MEK1/ERK and AKT/PKB pathways. Int J Mol Med.

[29] Takahashi K, Sone S, Saito S (1995). Granulocyte-macrophage colony-stimulating factor augments lymphokine-activated killer activity from pleural cavity mononuclear cells of lung cancer patients without malignant effusion. Jpn J Cancer Res.

[30] Bhattacharya P, Budnick I, Singh M (2015). Dual role of GM-CSF as a Pro-inflammatory and a regulatory cytokine: implications for immune therapy. J Interferon Cytokine Res.

[31] Brat DJ, Bellail AC, van Meir EG (2005). The role of interleukin-8 and its receptors in gliomagenesis and tumoral angiogenesis. Neuro-Oncol.

[32] Altorki NK, Markowitz GJ, Gao D (2019). The lung microenvironment: an important regulator of tumour growth and metastasis. Nat Rev Cancer.

[33] Yehya AHS, Asif M, Petersen SH (2018). Angiogenesis: managing the culprits behind tumorigenesis and metastasis. Medicina (Kaunas).

[34] Carpagnano GE, Spanevello A, Curci C (2007). IL-2, TNF-alpha, and leptin: local versus systemic concentrations in NSCLC patients. Oncol Res.

[35] Ryan BM, Pine SR, Chaturvedi AK, Caporaso N, Harris CC (2014). A combined prognostic serum interleukin-8 and interleukin-6 classifier for stage 1 lung cancer in the prostate, lung, colorectal, and ovarian cancer screening trial. J Thorac Oncol.

[36] Salem A, Mistry H, Backen A (2018). Cell death, inflammation, tumor burden, and proliferation blood biomarkers predict lung cancer radiotherapy response and correlate with tumor volume and proliferation imaging. Clin Lung Cancer.

[37] Matanić D, Beg-Zec Z, Stojanović D, Matakorić N, Flego V, Milevoj-Ribić F (2003). Cytokines in patients with lung cancer. Scand J Immunol.

[38] Vacca P, Martini S, Garelli V, Passalacqua G, Moretta L, Mingari MC (2013). NK cells from malignant pleural effusions are not anergic but produce cytokines and display strong antitumor activity on short-term IL-2 activation. Eur J Immunol.

[39] Pastuszak-Lewandoska D, Domańska-Senderowska D, Antczak A (2018). The expression levels of IL-4/IL-13/STAT6 signaling pathway genes and SOCS3 could help to differentiate the histopathological subtypes of non-small cell lung carcinoma. Mol Diagn Ther.

[40] Zaynagetdinov R, Sherrill TP, Gleaves LA (2015). Interleukin-5 facilitates lung metastasis by modulating the immune microenvironment. Cancer Res.

[41] Liu Z-H, Wang M-H, Ren H-J (2014). Interleukin 7 signaling prevents apoptosis by regulating bcl-2 and bax via the p53 pathway in human non-small cell lung cancer cells. Int J Clin Exp Pathol.

[42] Sanmamed MF, Perez-Gracia JL, Schalper KA (2017). Changes in serum interleukin-8 (IL-8) levels reflect and predict response to anti-PD-1 treatment in melanoma and non-small-cell lung cancer patients. Ann Oncol.

[43] Wang R, Lu M, Zhang J (2011). Increased IL-10 mRNA expression in tumor-associated macrophage correlated with late stage of lung cancer. J Exp Clin Cancer Res.

[44] Airoldi I, Di Carlo E, Cocco C (2009). IL-12 can target human lung adenocarcinoma cells and normal bronchial epithelial cells surrounding tumor lesions. PLoS ONE.

[45] Garris CS, Arlauckas SP, Kohler RH (2018). Successful anti-PD-1 cancer immunotherapy requires T Cell-dendritic cell crosstalk involving the cytokines IFN-γ and IL-12. Immunity.

[46] Joshi BH, Hogaboam C, Dover P, Husain SR, Puri RK (2006) Role of interleukin‐13 in cancer, pulmonary fibrosis, and other TH2‐type diseases. In: Interleukins. Elsevier; 479–504 10.1016/S0083-6729(06)74019-5 17027527

[47] Zhang Y, He S, Mei R (2018). miR-29a suppresses IL-13-induced cell invasion by inhibiting YY1 in the AKT pathway in lung adenocarcinoma A549 cells. Oncol Rep.

[48] Wu F, Xu J, Huang Q (2016). The role of interleukin-17 in lung cancer. Mediators Inflamm.

[49] Yan J, Smyth MJ, Teng MWL (2018). Interleukin (IL)-12 and IL-23 and their conflicting roles in cancer. Cold Spring Harb Perspect Biol.

