# Supplementary material for: Evaluation of cytokines in the tumor microenvironment of lung cancer using bronchoalveolar lavage fluid analysis
Source: Cancer Immunol Immunother. 2021 Jan 4;70(7):1867–76. doi: 10.1007/s00262-020-02798-z (PMC8195789; doi:10.1007/s00262-020-02798-z)
Supplement: Supplementary file 1 — Supplementary file1 (DOCX 255 KB) [file 262_2020_2798_MOESM1_ESM.docx]

*Table 6.* Cytokine levels in serum in lung cancer patients compared to healthy individuals

|  | Lung cancer | Healthy control | Univariate analysis  (p-value) | Multivariate analysis  (p-value) |
| --- | --- | --- | --- | --- |
|  |  |  |  |  |
| Fractalkine | 239.28  (183.11 – 304.15) | 131.84  (95.55 – 163.11) | <0.001 * | <0.001 * |
| GM-CSF | 53.98  (35.04 – 70.93) | 8.17  (6.47 – 13.94) | <0.001 * | <0.001 * |
| IFN-γ | 17.30  (10.08 – 32.89) | 4.91  (2.07 – 13.30) | <0.001 * | 0.023 * |
| IL-1b | 2.86  (1.95 – 4.00) | 0.30  (0.16 – 0.70) | <0.001 * | <0.001 * |
| IL-2 | 4.52  (2.35 – 6.61) | 1.61  (1.12 – 2.81) | <0.001 * | 0.116 * |
| IL-4 | 38.45  (23.79 – 52.58) | 7.68  (5.00 – 14.10) | <0.001 * | <0.001 * |
| IL-5 | 3.86  (2.44 – 5.65) | 3.57  (1.79 – 5.06) | 0.613 | 0.986 |
| IL-6 | 6.28  (3.80 – 9.13) | 2.25  (1.39 – 4.41) | <0.001 * | 0.596 |
| IL-7 | 15.15  (10.20 – 19.34) | 10.36  (8.84 – 14.33) | 0.008 * | 0.152 |
| IL-8 | 14.08  (9.76 – 20.82) | 7.34  (6.54 – 9.36) | <0.001 * | 0.007 * |
| Il-10 | 11.72  (8.33 – 17.09) | 8.30  (0.88 – 11.12) | 0.001 * | 0.713 |
| IL-12b | 4.99  (3.27 – 8.33) | 2.00  (0.98 – 3.20) | <0.001 * | <0.001 * |
| IL-13 | 8.90  (4.18 – 13.38) | 2.59  (0.95 – 5.90) | <0.001 * | 0.591 |
| IL-17a | 16.19  (10.29 – 32.45) | 2.82  (1.46 – 8.56) | <0.001 * | 0.003 * |
| IL-23 | 412.70  (203.21 – 741.72) | 178.27  (70.81 – 259.73) | <0.001 * | 0.003 * |
| TNF-α | 12.40  (10.40 – 16.58) | 4.61  (2.56 – 7.10) | <0.001 * | <0.001 * |

* = p < 0.05. Data are presented in median (IQR). Cytokine levels are denoted in pg/ml.

Abbreviations: BALF = bronchoalveolar lavage fluid, GM-CSF = granulocyte-macrophage colony-stimulating factor, IFN-γ = interferon gamma, IL = interleukin, TNF-α = tumor necrosis factor alpha

*Table 7.* Cytokine levels in serum in lung cancer patients compared to other lung diseases.

|  | Lung cancer | Other lung diseases | Univariate analysis  (p-value) | Multivariate analysis  (p-value) |
| --- | --- | --- | --- | --- |
|  |  |  |  |  |
| Fractalkine | 239.28  (183.11 – 304.15) | 177.22  (105.28 – 320.84) | 0.072 | 0.365 |
| GM-CSF | 53.98  (35.04 – 70.93) | 21.12  (10.67 – 79.78) | 0.012 * | 0.485 |
| IFN-γ | 17.30  (10.08 – 32.89) | 9.40  (4.33 – 22.96) | 0.032 * | 0.110 |
| IL-1b | 2.86  (1.95 – 4.00) | 1.10  (0.35 – 3.14) | 0.001 * | 0.144 |
| IL-2 | 4.52  (2.35 – 6.61) | 2.85  (1.58 – 5.15) | 0.064 | 0.572 |
| IL-4 | 38.45  (23.79 – 52.58) | 17.31  (7.52 – 48.50) | 0.009 * | 0.559 |
| IL-5 | 3.86  (2.44 – 5.65) | 3.79  (2.25 – 6.38) | 0.958 | 0.683 |
| IL-6 | 6.28  (3.80 – 9.13) | 4.51  (2.49 – 8.95) | 0.087 | 0.574 |
| IL-7 | 15.15  (10.20 – 19.34) | 13.44  (9.43 – 20.88) | 0.969 | 0.719 |
| IL-8 | 14.08  (9.76 – 20.82) | 9.39  (6.29 – 18.02) | 0.028 * | 0.160 |
| Il-10 | 11.72  (8.33 – 17.09) | 9.57  (3.96 – 16.16) | 0.113 | 0.172 |
| IL-12b | 4.99  (3.27 – 8.33) | 3.54  (1.47 – 6.54) | 0.064 | 0.588 |
| IL-13 | 8.90  (4.18 – 13.38) | 3.93  (1.70 – 8.14) | 0.015 * | 0.438 |
| IL-17a | 16.19  (10.29 – 32.45) | 10.01  (4.05 – 21.73) | 0.007 * | 0.070 |
| IL-23 | 412.70  (203.21 – 741.72) | 340.26  (131.06 – 472.38) | 0.157 | 0.100 |
| TNF-α | 12.40  (10.40 – 16.58) | 10.96  (5.86 – 17.74) | 0.235 | 0.523 |

* = p < 0.05. Data are presented in median (IQR). Cytokine levels are denoted in pg/ml.

Abbreviations: BALF = bronchoalveolar lavage fluid, GM-CSF = granulocyte-macrophage colony-stimulating factor, IFN-γ = interferon gamma, IL = interleukin, TNF-α = tumor necrosis factor alpha

# Appendix

For better visualization we included figures showing the measured cytokine levels in all three groups for each cytokine as well as for serum and BALF.

## Fractalkine

*Figure 13.1.1.* fractalkine in serum


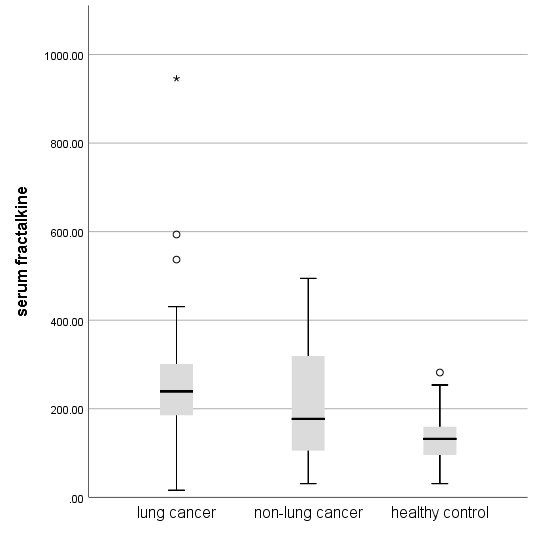


Cytokine levels are denoted in pg/ml.

*Figure 13.1.2.* fractalkine in BALF


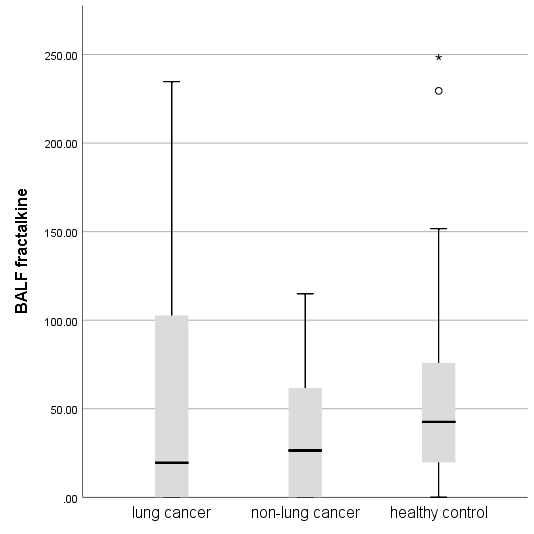


Cytokine levels are denoted in pg/ml.

Abbreviations: BALF = bronchoalveolar lavage fluid

## GM-CSF

*Figure 13.2.1* GM-CSF in serum


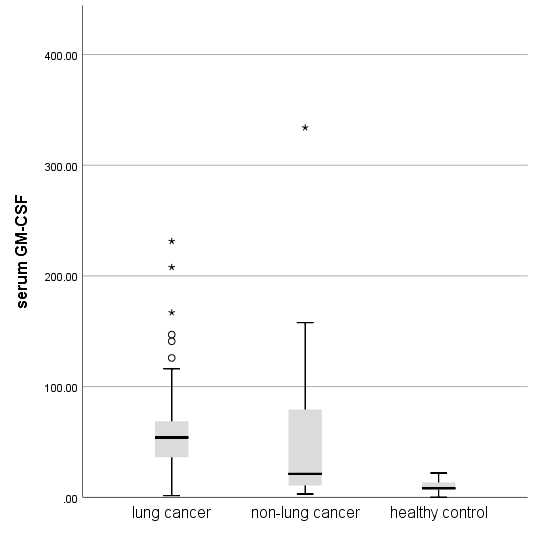


Cytokine levels are denoted in pg/ml.

Abbreviations: GM-CSF = granulocyte-macrophage colony-stimulating factor

*Figure 13.2.2* GM-CSF in BALF


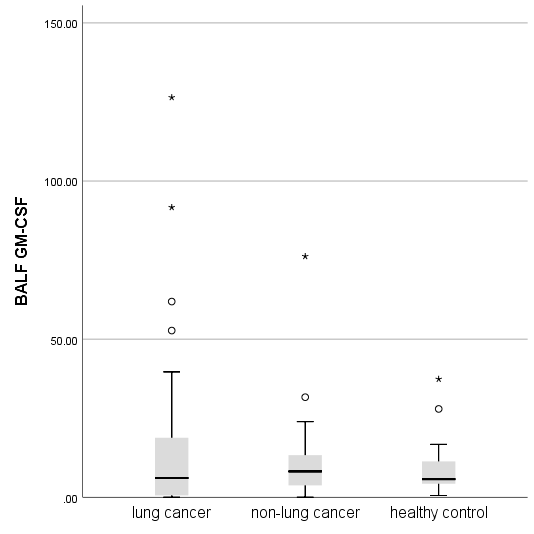


Cytokine levels are denoted in pg/ml.

Abbreviations: BALF = bronchoalveolar lavage fluid, GM-CSF = granulocyte-macrophage colony-stimulating factor

## IFN- γ

*Figure 13.3.1* IFN- γ in serum


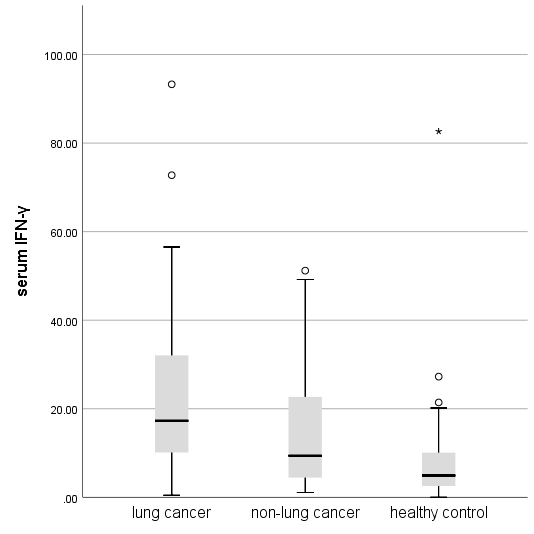


Cytokine levels are denoted in pg/ml.

Abbreviations: IFN-γ = interferon gamma

*Figure 13.3.2* IFN- γ in BALF


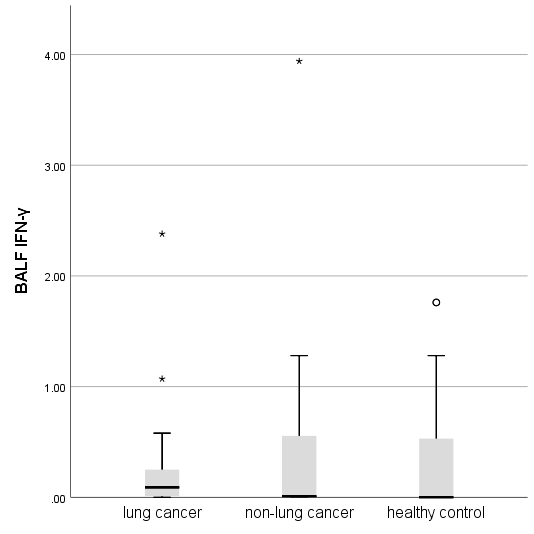


Cytokine levels are denoted in pg/ml.

Abbreviations: BALF = bronchoalveolar lavage fluid, IFN-γ = interferon gamma

## IL-1b

*Figure 13.4.1* IL-1b in serum


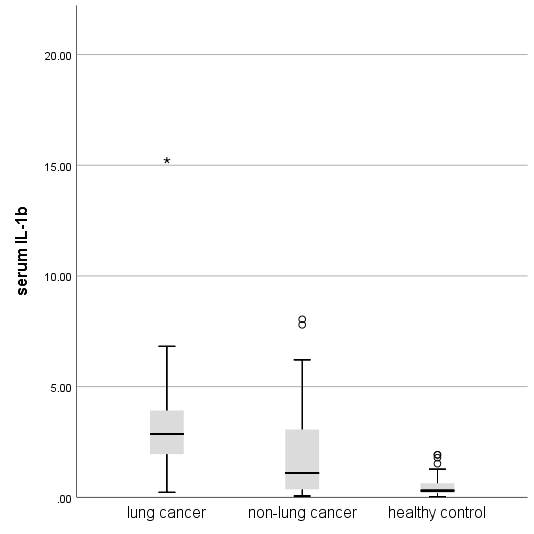


Cytokine levels are denoted in pg/ml.

Abbreviations: IL = interleukin

*Figure 13.4.2* IL-1b in BALF


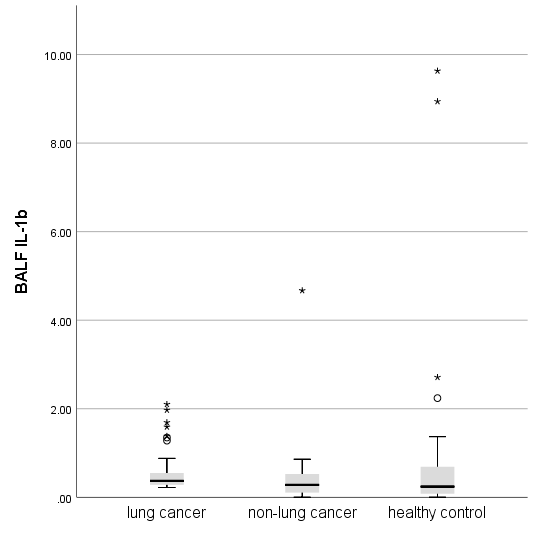


Cytokine levels are denoted in pg/ml.

Abbreviations: BALF = bronchoalveolar lavage fluid, IL = interleukin

## IL-2

*Figure 13.5.1* IL-2 in serum


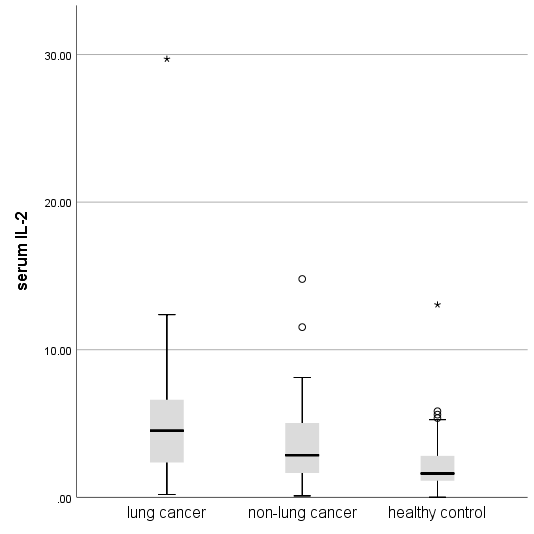


Cytokine levels are denoted in pg/ml.

Abbreviations: IL = interleukin

*Figure 13.5.2* IL-2 in BALF


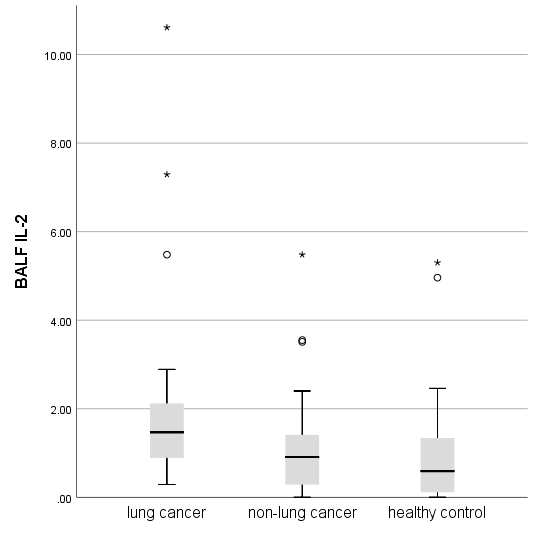


Cytokine levels are denoted in pg/ml.

Abbreviations: BALF = bronchoalveolar lavage fluid, IL = interleukin

## IL-4

*Figure 13.6.1* IL-4 in serum


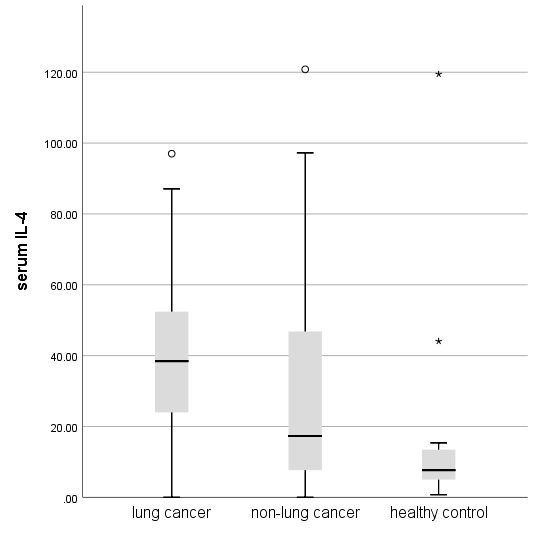


Cytokine levels are denoted in pg/ml.

Abbreviations: IL = interleukin

*Figure 13.6.2* IL-4 in BALF


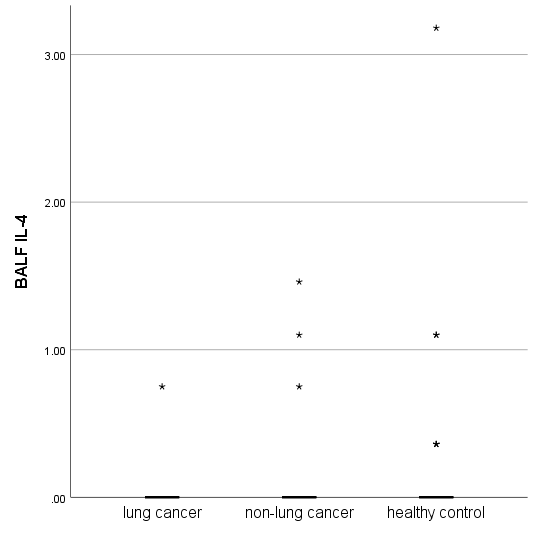


Cytokine levels are denoted in pg/ml.

Abbreviations: BALF = bronchoalveolar lavage fluid, IL = interleukin

## IL-5

*Figure 13.7.1* IL-5 in serum


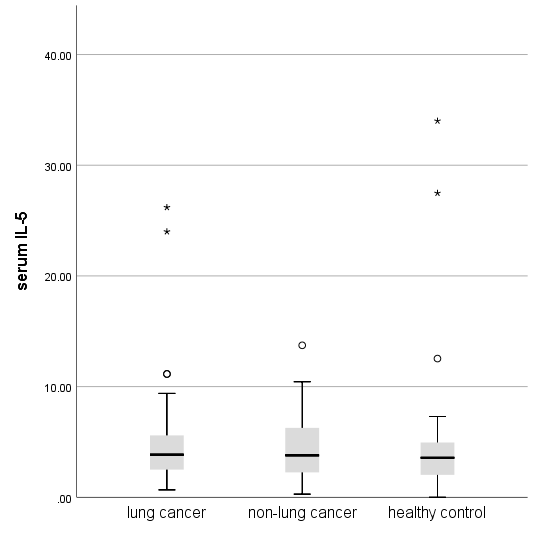


Cytokine levels are denoted in pg/ml.

Abbreviations: IL = interleukin

*Figure 13.7.2* IL-5 in BALF


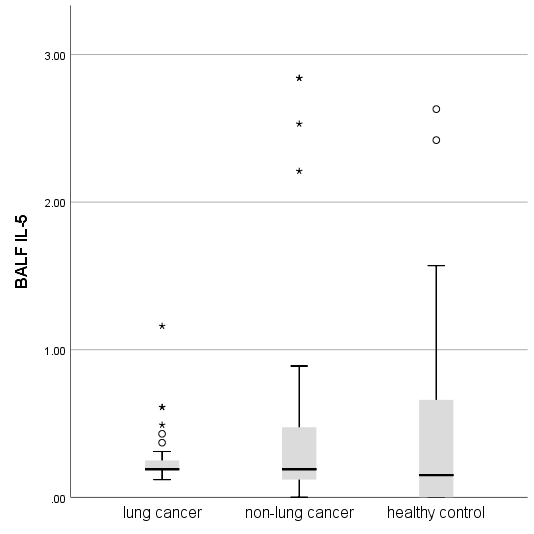


Cytokine levels are denoted in pg/ml.

Abbreviations: BALF = bronchoalveolar lavage fluid, IL = interleukin

## IL-6

*Figure 13.8.1* IL-6 in serum


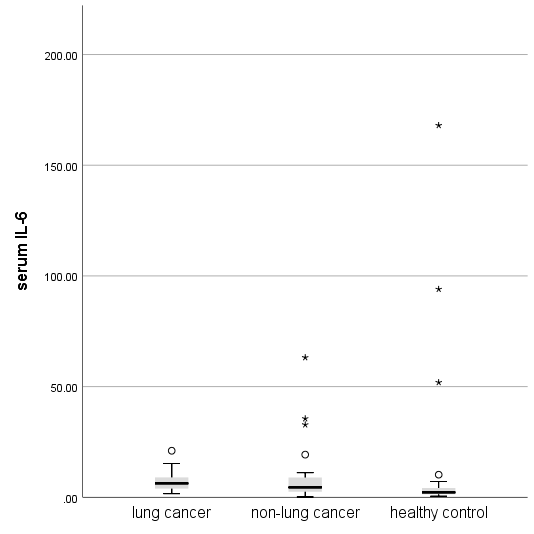


Cytokine levels are denoted in pg/ml.

Abbreviations: IL = interleukin

*Figure 13.8.2* IL-6 in BALF


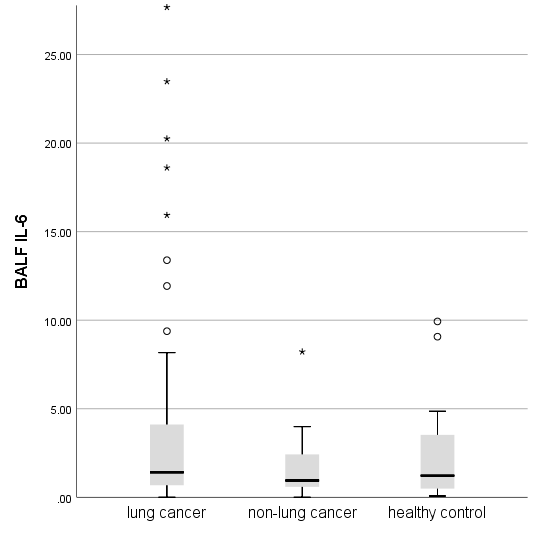


Cytokine levels are denoted in pg/ml.

Abbreviations: BALF = bronchoalveolar lavage fluid, IL = interleukin

## IL-7

*Figure 13.9.1* IL-7 in serum


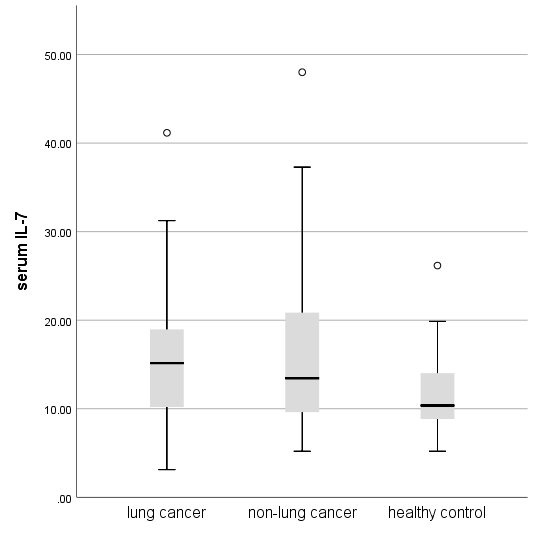


Cytokine levels are denoted in pg/ml.

Abbreviations: IL = interleukin

*Figure 13.9.2* IL-7 in BALF


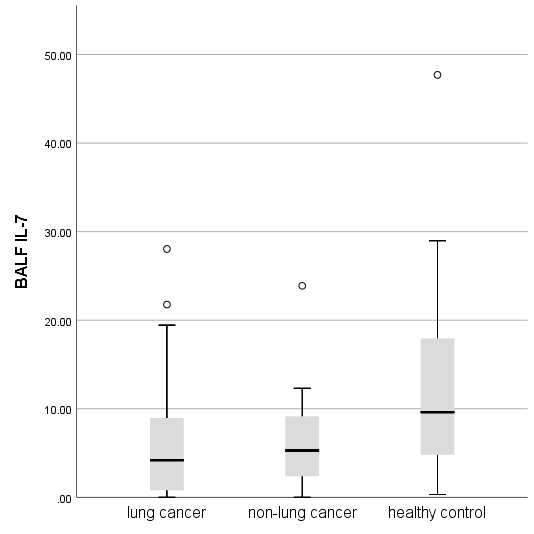


Cytokine levels are denoted in pg/ml.

Abbreviations: BALF = bronchoalveolar lavage fluid, IL = interleukin

## IL-8

*Figure 13.10.1* IL-8 in serum


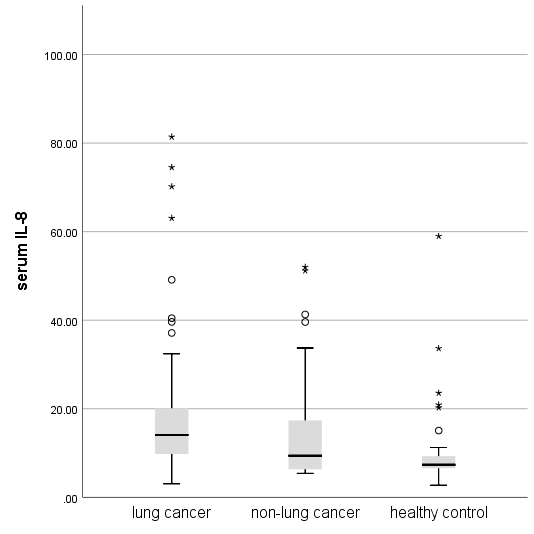


Cytokine levels are denoted in pg/ml.

Abbreviations: IL = interleukin

*Figure 13.10.2* IL-8 in BALF


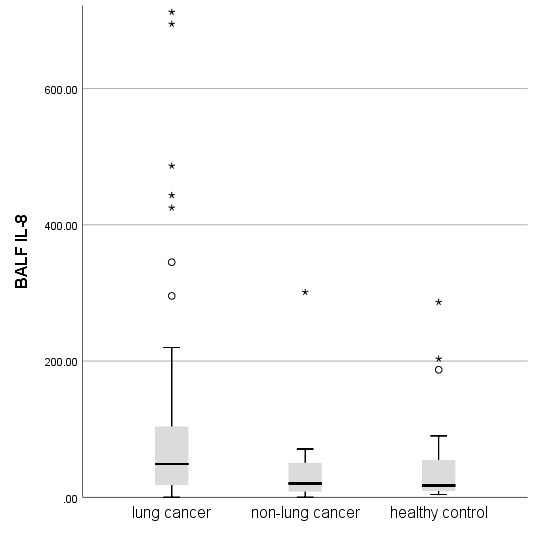


Cytokine levels are denoted in pg/ml.

Abbreviations: BALF = bronchoalveolar lavage fluid, IL = interleukin

## IL-10

*Figure 13.11.1* IL-10 in serum


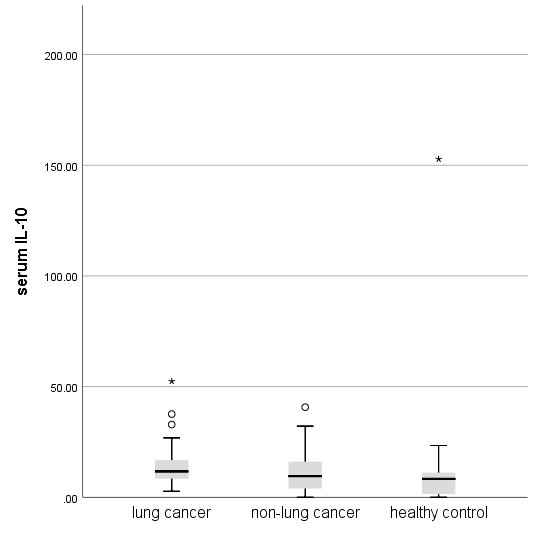


Cytokine levels are denoted in pg/ml.

Abbreviations: IL = interleukin

*Figure 13.11.2* IL-10 in BALF


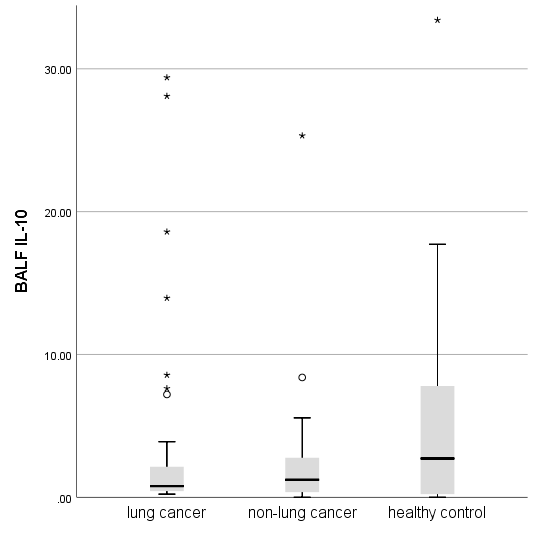


Cytokine levels are denoted in pg/ml.

Abbreviations: BALF = bronchoalveolar lavage fluid, IL = interleukin

## IL-12b

*Figure 13.12.1* IL-12b in serum


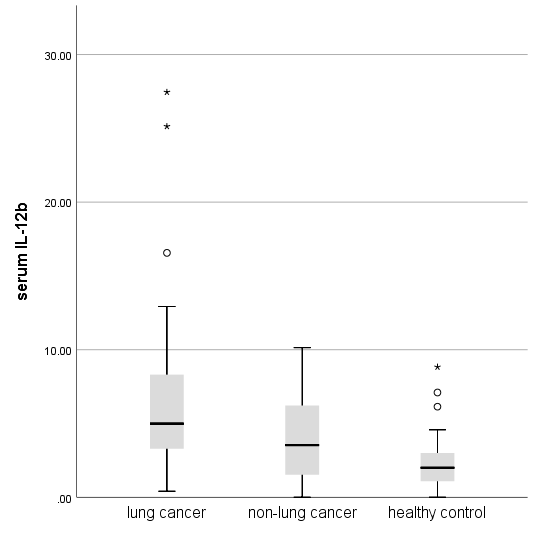


Cytokine levels are denoted in pg/ml.

Abbreviations: IL = interleukin

*Figure 13.12.2* IL-12b in BALF


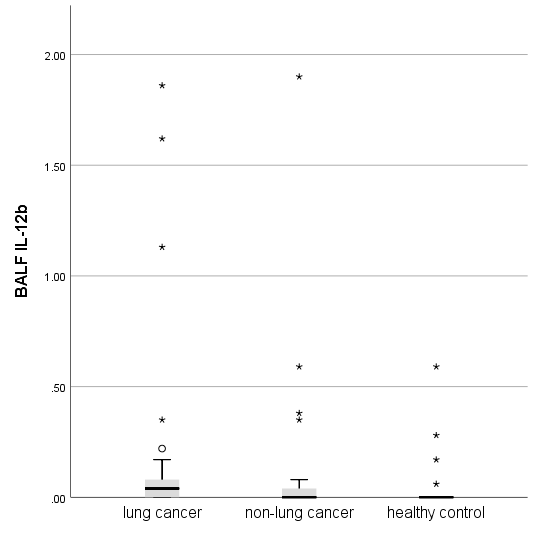


Cytokine levels are denoted in pg/ml.

Abbreviations: BALF = bronchoalveolar lavage fluid, IL = interleukin

## IL-13

*Figure 13.13.1* IL-13 in serum


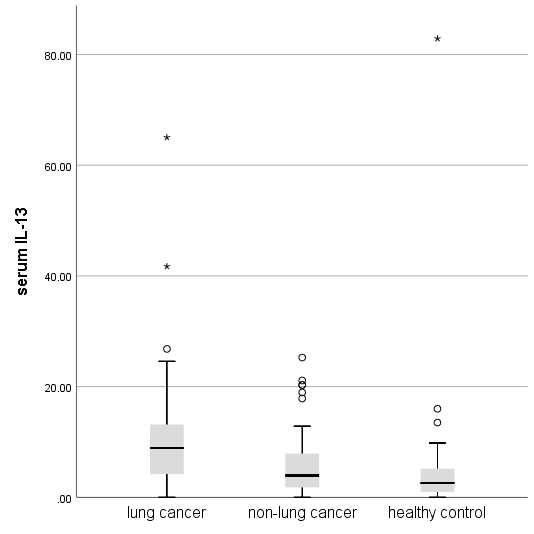


Cytokine levels are denoted in pg/ml.

Abbreviations: IL = interleukin

*Figure 13.13.2* IL-13 in BALF


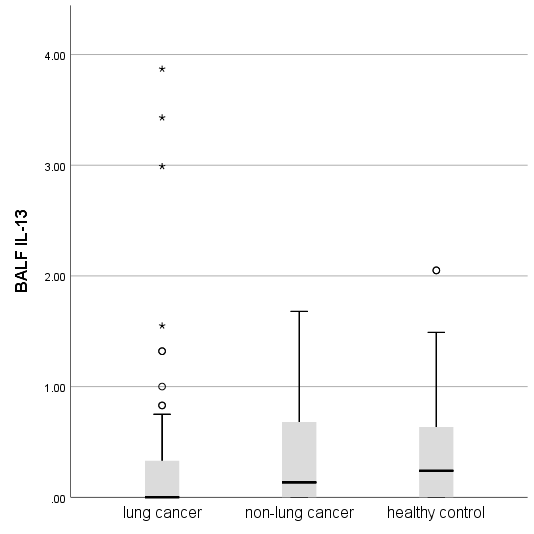


Cytokine levels are denoted in pg/ml.

Abbreviations: BALF = bronchoalveolar lavage fluid, IL = interleukin

## IL-17a

*Figure 13.14.1* IL-17a in serum


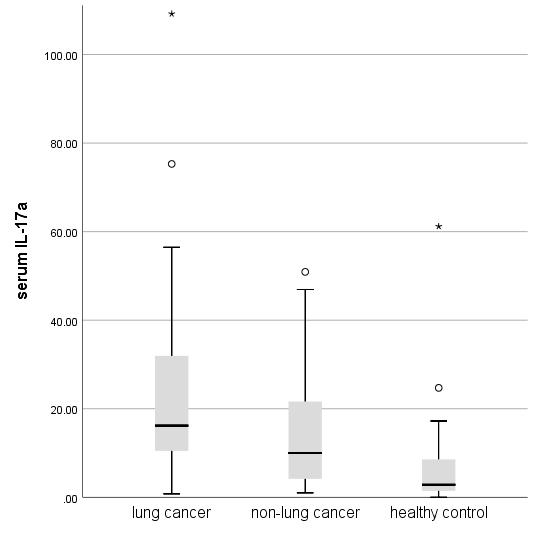


Cytokine levels are denoted in pg/ml.

Abbreviations: IL = interleukin

*Figure 13.14.1* IL-17a in BALF


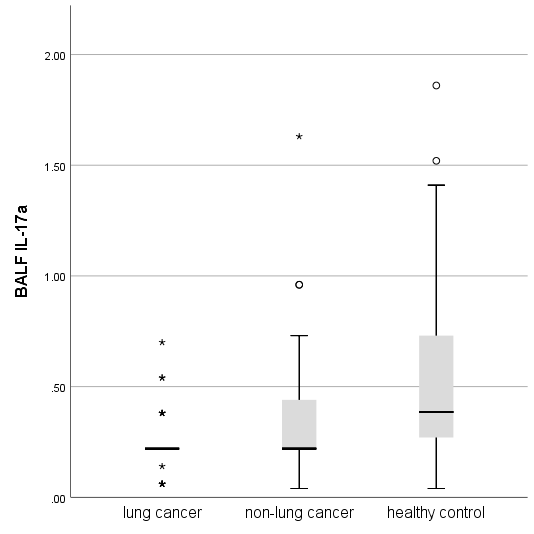


Cytokine levels are denoted in pg/ml.

Abbreviations: BALF = bronchoalveolar lavage fluid, IL = interleukin

## IL-23

*Figure 13.15.1* IL-23 in serum


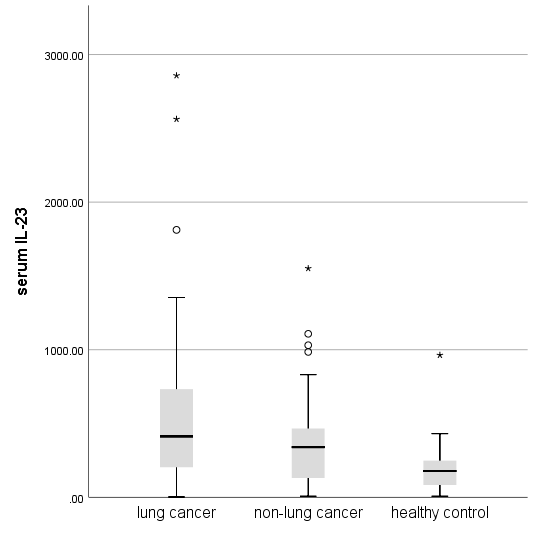


Cytokine levels are denoted in pg/ml.

Abbreviations: IL = interleukin

*Figure 13.15.2* IL-23 in BALF


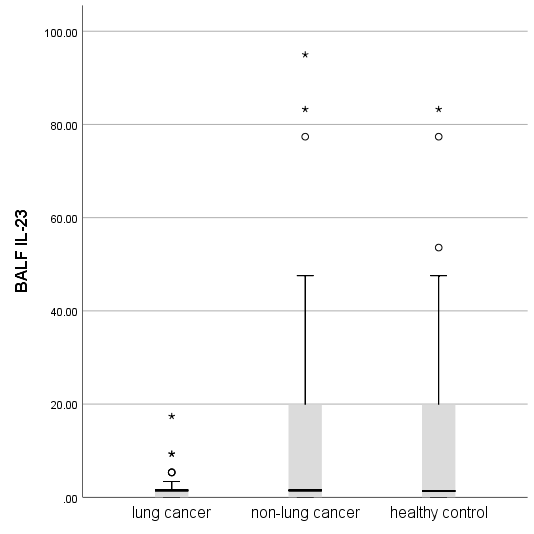


Cytokine levels are denoted in pg/ml.

Abbreviations: BALF = bronchoalveolar lavage fluid, IL = interleukin

## TNF- α

*Figure 13.16.1* TNF- α in serum


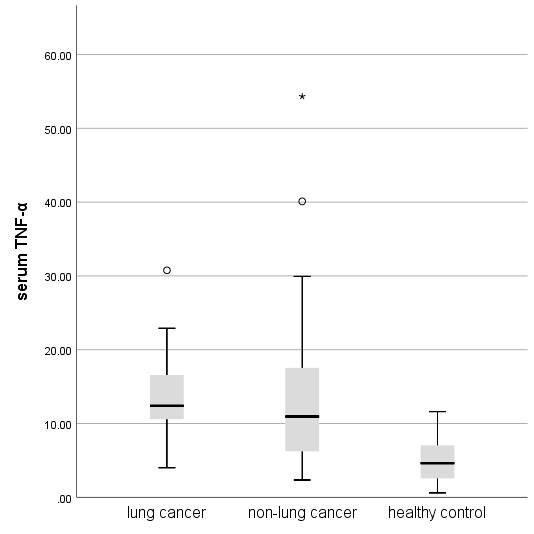


Cytokine levels are denoted in pg/ml.

Abbreviations: TNF-α = tumor necrosis factor alpha

*Figure 13.16.2* TNF- α in BALF


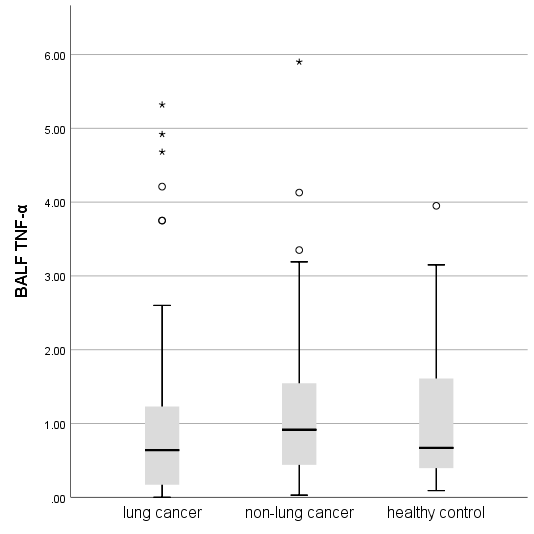


Cytokine levels are denoted in pg/ml.

Abbreviations: BALF = bronchoalveolar lavage fluid, TNF-α = tumor necrosis factor alpha
